# Automatic segmentation of paravertebral muscles in abdominal CT scan by U-Net: The application of data augmentation technique to increase the Jaccard ratio of deep learning

**DOI:** 10.1097/MD.0000000000027649

**Published:** 2021-11-05

**Authors:** Kuen-Jang Tsai, Chih-Chun Chang, Lun-Chien Lo, John Y. Chiang, Chao-Sung Chang, Yu-Jung Huang

**Affiliations:** a Department of Surgery, E-Da Cancer Hospital, Taiwan; b College of Medicine, I-Shou University, Kaohsiung, Taiwan; c Department of Computer Science and Engineering, National Sun Yat-sen University, Kaohsiung, Taiwan; d School of Chinese Medicine, China Medical University, Taichung, Taiwan; e Department of Chinese Medicine, China Medical University Hospital, Taichung, Taiwan; f Department of Healthcare Administration and Medical Informatics, Kaohsiung Medical University, Kaohsiung, Taiwan; g Department of Medical Imaging and Radiological Sciences, Kaohsiung Medical University, Kaohsiung, Taiwan; h Department of Hematology/Oncology, E-Da Cancer Hospital, School of Medicine for International Students, I-Shou University, Kaohsiung, Taiwan; i Department of Electronic Engineering, I-Shou University, Kaohsiung, Taiwan.

**Keywords:** cross-validation, medical image segmentation, paravertebral muscles, sarcopenia, U-Net

## Abstract

Sarcopenia, characterized by a decline of skeletal muscle mass, has emerged as an important prognostic factor for cancer patients. Trunk computed tomography (CT) is a commonly used modality for assessment of cancer disease extent and treatment outcome. CT images can also be used to analyze the skeletal muscle mass filtered by the appropriate range of Hounsfield scale. However, a manual depiction of skeletal muscle in CT scan images for assessing skeletal muscle mass is labor-intensive and unrealistic in clinical practice. In this paper, we propose a novel U-Net based segmentation system for CT scan of paravertebral muscles in the third and fourth lumbar spines. Since the number of training samples is limited (i.e., 1024 CT images only), it is well-known that the performance of the deep learning approach is restricted due to overfitting. A data augmentation strategy to enlarge the diversity of the training set to boost the performance further is employed. On the other hand, we also discuss how the number of features in our U-Net affects the performance of the semantic segmentation. The efficacies of the proposed methodology based on w/ and w/o data augmentation and different feature maps are compared in the experiments. We show that the Jaccard score is approximately 95.0% based on the proposed data augmentation method with only 16 feature maps used in U-Net. The stability and efficiency of the proposed U-Net are verified in the experiments in a cross-validation manner.

## 1 Introduction

Reduced skeletal muscle mass (sarcopenia) is associated with loss of physical function, disability,^[1]^ risk of falls and fracture,^[2]^ and increased length of hospital stay.^[3]^ Sarcopenia was also strongly correlated with treatment outcomes in patients with cancer.^[4]^ Computed tomography (CT) is a widely-used examination tool in the treatment of various cancer diseases. The majority of cancer diseases mandate CT scanning of the abdomen, pelvis, or chest for a pre-treatment assessment. Each kind of human body composition has unique radiation attenuation, expressed as Hounsfield units (HU), in CT scans so that CT images have very high precision and specificity for different tissue in the human body.^[5]^ The CT scan is frequently used to review the change of skeletal muscle area and density for monitoring the patient's health status.^[6]^ Manual depiction of body compartments on the computer screen is based solely on the judgement of physicians’ “surgical intuition” or “eyeball test.” This practice is not only laborious but also unrealistic in clinical practice and has long been criticized for its low inter-rater and intra-rater consistencies. Therefore, the urgent need for automatic segmentation of the body compartment assisted by artificial intelligence (AI) is emerging.

Paravertebral skeletal muscle areas, that is, psoas muscle, lumborum quadratus, and para-spinal muscle, at the third and fourth lumbar spine levels derived from abdominal CT scan have been shown to be associated with treatment outcomes in patients with various cancer diseases. The patients with lower muscle area and density usually will have worse treatment outcomes.^[7–9]^

Recently, the clinical applications of deep learning-based medical images interpretation including organ and body compartment segmentation, disease detection, and assessment of response to treatment have gained increasing interests.^[10–13]^ The deep learning approach, or so-called AI, is a data-driven learning technique. In other words, AI techniques take the essential features themselves from data without any guidance. Automatic segmentation of the boundaries of an organ or lesion is a crucial AI application for reducing the burden of radiologists. It also provides vital information on the functional performance of tissues and organs, and disease extent.^[14]^ An AI-driven model to automatically interpret CT images may greatly facilitate clinical practice and increase diagnostic consistency.

U-Net is a well-known semantic segmentation technique for the medical image as well as is widely utilized in recognizing cells or organs.^[15]^ U-Net employs encoder-decoder network architecture to tackle the multiresolution features aggregation issue. Since the number of the parameters of a typical U-Net is relatively small, the requirement of the number of training samples is fewer compared to other state-of-the-art semantic segmentation networks, such as a fully convolutional network, DeepLabv3, Mask-RCNN.^[16,17]^ U-Net is composed of a convolution neural network (feature extraction), downsampling (size reduction and feature retainment), up-sampling (size recovery), and finally, generation of the feature map. Since data labeling is a very time consuming and tedious work, the collection of a sufficient and relatively large-scale image dataset for AI training is challenging. To effectively overcome this issue, U-Net is adopted as our baseline, and a data augmentation is then proposed to effectively increase the diversities of the collected and small-scale training set. In this fashion, we can achieve a significant improvement in terms of the Jaccard ratio (i.e., intersection over union). Even though U-Net has been widely used on CT image segmentation, for example, lung, kidney, kidney tumor, chest, etc, the application of U-Net based segmentation system specifically for CT scan of paravertebral muscles in the third and fourth lumbar spines, to the best of our knowledge, remains regrettably void. The challenges posed by this study of paravertebral muscles include the number of CT images available, scanner type and contrast phase (venous, portal venous, arterial).^[10,15]^

## 2 Materials and methods

**Figure 1:**
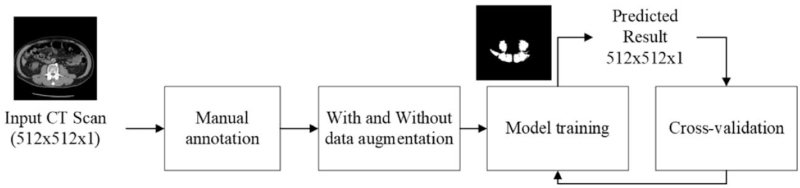
The input CT image size is 512 × 512 × 1. After being labeled by the doctor, the dataset is split into 2 paths: one with data augmentation and the other without. After training and comparing the mIOU of the 2, the output result after training is a prediction mask of 512 × 512 × 1. CT = computed tomography, mIOU = mean intersection over union.

The initial input of the study is a grayscale image with a size of 512 × 512 × 1, and a mask of 512 × 512 × 1 contains 2 classes of images, where the pixel value 0 indicates the background and 1 the muscle. There are 1024 intravenous contrast medium enhanced CT images in total. The collected dataset is partitioned into the training, validation, and testing sets to verify the performance for finding the best model of the proposed U-Net. We also verify the necessity of whether the data augmentation is useful or not in improving the segmentation performance. For efficiency and accuracy, the training process uses the same verification set, and test set stated previously. The size of the predicted map is the same as that of the ground truth (i.e., 512 × 512 × 1). Mean intersection over union (mIOU) is adopted to verify the performance of the proposed method (Fig. 1).

### 2.1 Dataset

**Figure 2:**
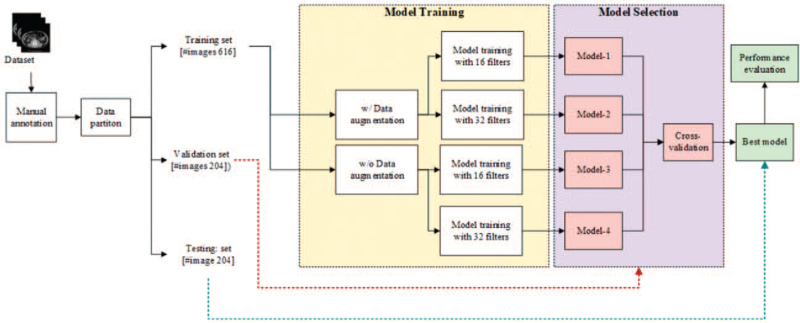
System flow. The CT scans of gastric cancer patients serve as the data source. The regions of interest in CT scan images are labeled manually as the ground truth. The dataset containing 1024 images was further randomly partitioned into 3 disjoint groups: training set with 616 images, validation with 204 images, and the rest are used to form the testing set. Data augmentation of the training images is performed to obtain the augmented training set. Four models were established based on 16 or 32 filters in the proposed U-Net with and without data augmentation. The best model was obtained by the accuracy and stability based on the validation set. CT = computed tomography.

The data were collected retrospectively for abdominal CT examinations of 264 patients of gastric cancer receiving surgery in E-Da and E-Da Cancer hospital between 2007 and 2017. The first 2 cross-sectional images at the level of the third and fourth lumbar vertebra from the cranial-caudal direction, that is, 4 images per subject, were acquired. Among these 1056 images, 32 slices (8 patients) were excluded due to poor image quality. Therefore, we have a total of 1024 images to form our CT dataset. Table 1 summarizes patient characteristics of the dataset including image slice thickness, pixel resolution, patient age, sex, as well as scanner type and contrast phase. The CT dataset was further randomly partitioned into 3 disjoint groups on a per-patient basis in the ratio of 6:2:2, in which the training set is composed of 154 patents, that is, 616 images, the validation set contains 51 patients, that is, 204 images, and the rest are formed as the test set. This splitting scheme is performed on the per-patient basis to make sure 4 images from the same subject will not exist in both the training and testing/or validation and testing sets. The proposed CT-aware data augmentation (CTDA) is then performed on the training set to obtain the augmented training set with 3080 (616 × 5) images. We create 4 models based on the proposed U-Net and CTDA to seek the best model. Finally, the standard cross-validation (CV) process is made based on the validation set to assess the performance of these 4 models. Based on the observed performance, the best model in terms of accuracy and stability is then selected as the final model. The processing flow is schematically depicted in Figure 2.

**Table 1:** Information of patients and CT techniques.

Patient age	34–93 year old (mean 67.2 ± 10.9)
Sex	Male: 163, female: 101
Slice thickness	5 mm
Pixel resolution	512 × 512 pixel
Scanner type	GE Bright Speed
Contrast phase	Venous phase

CT = computed tomography.

### 2.2 Manual segmentation

**Figure 3:**
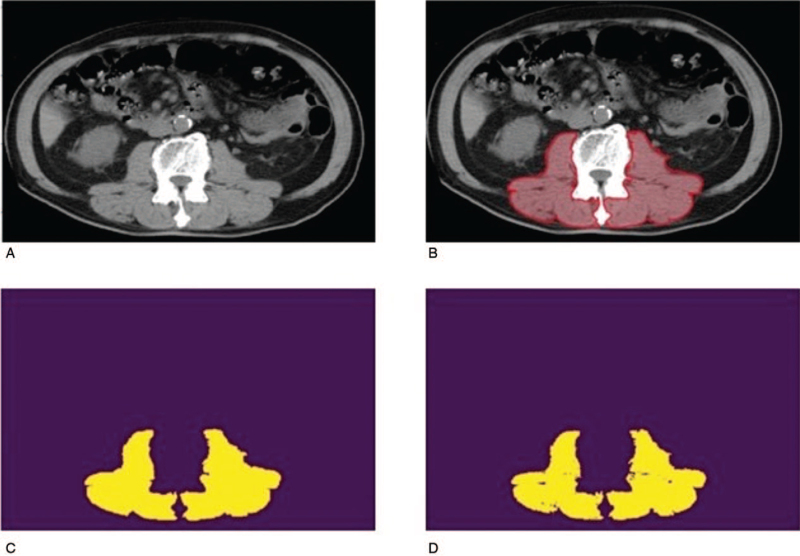
(A) Original image of a CT scan. (B) Manual segmentation of regions of interest, including bilateral psoas muscle, lumborum quadratus, and paraspinal muscle. (C) Mask of the image after manual segmentation. (D) Ground truth after the HU filtering. CT = computed tomography, HU = Hounsfield units.

Manual labeling of the circumferences of regions of interest (ROI), including bilateral psoas muscle, lumborum quadratus, and paraspinal muscle, in each image was performed on the original image (Fig. 3A) by an experienced surgeon as accurately as possible with the in-house software, as shown in Figure 3B. After acquiring the ROI mask of manual segmentation, as shown in Figure 3C, the ground truth is obtained by applying HU filter with the value of skeletal muscle ranging from –29 HU to +150 HU^[18]^ to the segmented maps (Fig. 3D).

### 2.3 Data augmentation

Many skills have been proposed for data augmentation in deep learning techniques for generic images. However, unlike the generic images, the content, and the contextual information in the CT scans are significantly different, leading to a fact that the conventional data augmentation might be inefficient and ineffective. Based on the essential experiences of the experienced surgeons, the data augmentation operation should be carefully designed to avoid destroying the intrinsic features of the CT scans. In this study, the affine transformations, including shift, horizontal flipping, and rotation, were applied to increase the size of the dataset. The shift ratio was set to 5% of the image size in either vertical or horizontal direction. The rotation angle range is randomly selected from ±20° for simulating the patients’ body position variations while the abdominal CT scans were acquired.^[19,20]^ The variables for each of the aforementioned affine transformations were selected randomly within each specified range.

### 2.4 U-Net

In this study, U-Net architecture is utilized to design our baseline model. In general, U-Net contains an encoder and a decoder to extract semantic features from CT scans and aggregate the multiscale features into fine resolution ones. It is well-known that the U-Net is an effective way to tackle the issues commonly encountered in medical information processing. In our U-Net, every convolutional layer in the convolution neural network is composed of 2 3 × 3 filters sharing the same padding size. The batch size and the initial channel number are 4 and 32, respectively. The learning rate sets to 0.0001 with Adam optimizer for the initial training hyper-parameters. The Leaky Rectified Linear Unit is used as the activation function.^[21]^ The input image is digital imaging and communications in medicine-formatted with one-channel size 512 × 512 pixels.

**Figure 4:**
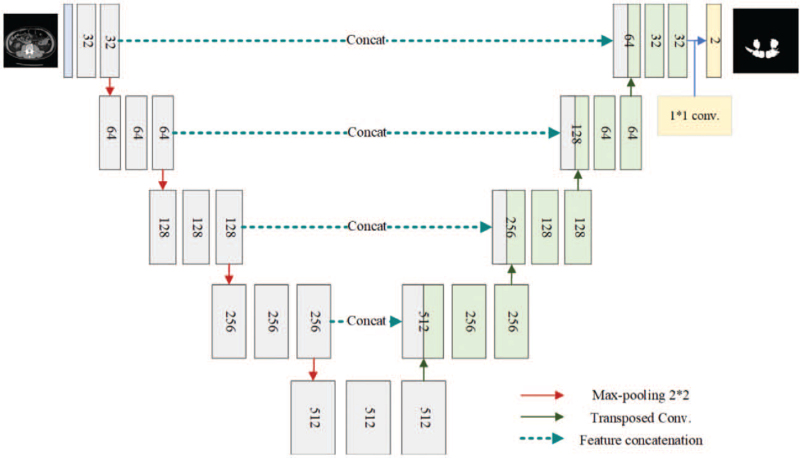
The network architecture of the proposed U-Net.

Let n_base_ denote the number of the filters in the first convolutional layer in our U-Net model, as depicted in Figure 4, the number of the filters of the i_th_ convolutional layer is given by


$${\text{n}}_{\text{base}}^{\text{i}}={\text{n}}_{\text{base}}\times 2^{\text{i}-1},i=1,2,\ldots \text{L}.$$

where *L* is the number of total layers of our U-Net model. In this study, n_base_ is set to 16 and 32, respectively. As the number of the filters increases, the performance of the proposed method should be improved accordingly, while the complexity would be increased as well. In this study, we verify this by extensive experiments by using CV to find the best setting for n_base_. A 2 × 2 max-pooling operation with a strided size of 2 is performed at the end of each convolutional layer to reduce the spatial resolution in the encoder side, as well as a 2 × 2 transposed convolution is applied on the decoder side to improve the spatial resolution progressively.^[22]^ Afterward, the output is connected with a 1 × 1 convolution operation to fuse the feature maps to be a class-wise representation. Finally, the Softmax activation function is used to normalize the response values of the class-wise feature maps. By simply applying the maximum operation on the class-wise feature maps along with channel-direction, the predicted segmentation map can be obtained. The structure of the model is shown in Figure 4. In the training phase, the early stop policy is used based on mIOU metric. The number of epochs of the training process is 1000. Both the number of early stop strategy and the patience value of the learning decay policy is 100 iterations. In general, each training takes about 300 to 400 epochs to converge.

### 2.5 Cross-validation

In this study, the holdout CV is adopted. Holdout CV randomly partitioned the dataset into 3 disjoint parts: training (60%), validation (20%), and testing (20%) datasets. In order to obtain a stable result, the holdout CV is repeated 3 independent rounds.^[23]^ Each round will train our model based on the training set and verify the performance (i.e., Jaccard score) on the testing set. Noted that the training/validation/testing datasets in each round are randomly sampled from the original dataset, leading a fact that the training sets of different rounds are different from each other. The final Jaccard score is measured by unweighted averaging on the 3 individual measures.

## 3 Results

A personal computer equipped with Nvidia Tesla V100, Intel(R) Xeon(R) CPU E5-2650 v4 @ 2.20 GHz, and 192 GB system RAM is used to evaluate the performance of the proposed method. The experiments are designed deliberately from 2 perspectives: non-augmented vs augmented dataset and the number of features. In order to understand the benefits of data augmentation, we verify the effectiveness of the proposed U-Net based on the training set with and without data augmentation for 16 and 32 feature maps.

**Figure 5:**
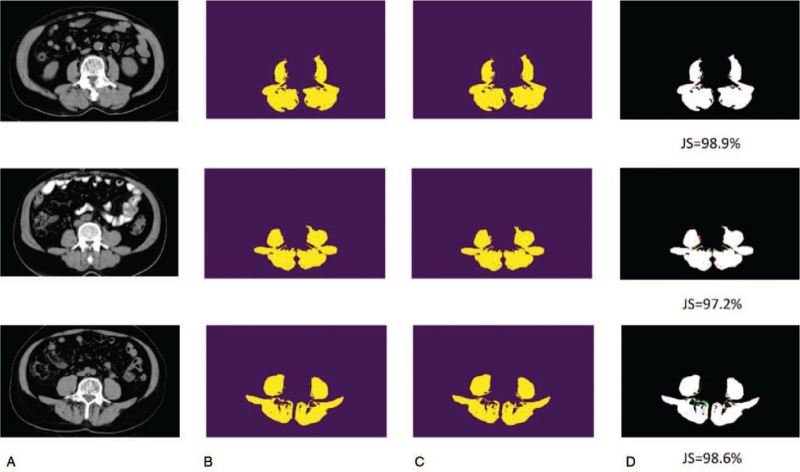
U-Net prediction result. The cases with a higher Jaccard score. (A) Input: the original CT scan. (B) Ground truth: labeled images. (C) Prediction result. (D) The white region corresponds to true-positive, green one false-negative, and red one false-positive. CT = computed tomography.

**Figure 6:**
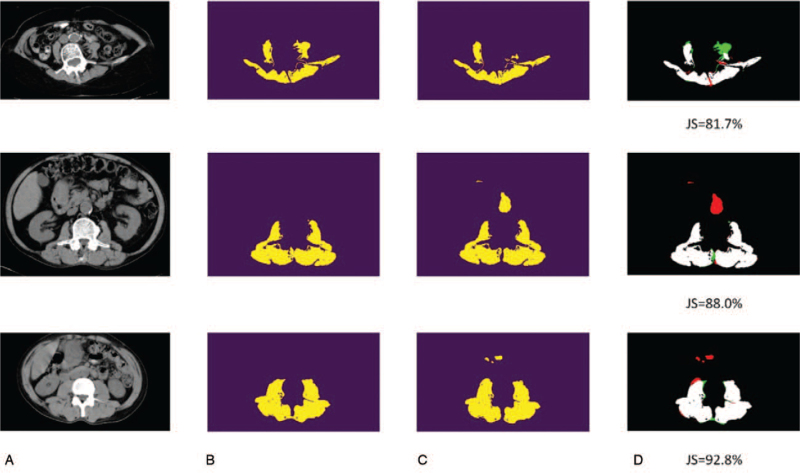
U-Net prediction result. The challenging cases with lower Jaccard score. (A) Input: the original CT scan. (B) Ground truth: labeled images. (C) Prediction result. (D) The white region corresponds to true-positive, the green one false-negative, and the red one false-positive. CT = computed tomography.

As shown in Table 1 and Figures 5 and 6, it is surprising that the best model is based on 16 filters as the initial number of filters. Since the number of the training set is extremely limited, compared to the generic images, it is reasonable that the parameters of the deep neural network should be smaller to avoid the issues caused by overfitting. As a result, it is recommended that the n_base_ = 16 is the suggested setting for muscle segmentation of CT scans. In contrast, the proposed CTDA shows the performance improvement for both 16 and 32 filters for the initial convolutional layer in our U-Net. The ground truth is obtained by applying HU filter with the value of skeletal muscle ranging from –29 HU to +150 HU^[18]^ to the segmented maps (Fig. 3D). The U-Net does not recognize the muscle area according to the HU but is trained by the HU filtered image of manually segmented ROI. As depicted in Table 1, our U-Net can achieve 95.0% in terms of averaged mIOU in the model of 16 features with data augmentation. We also demonstrate cases with higher and lower Jaccard scores, including the original, labeled, and prediction CT images with false-positive and false-negative regions highlighted, are shown in Figures 5 and 6, respectively. Although some false-positive or false-negative regions are shown in Figures 5 and 6, it is remarkable that the integrity of true-positive regions remains intact, implying that the information is sufficient for assisting diagnosis.

**Figure 7:**
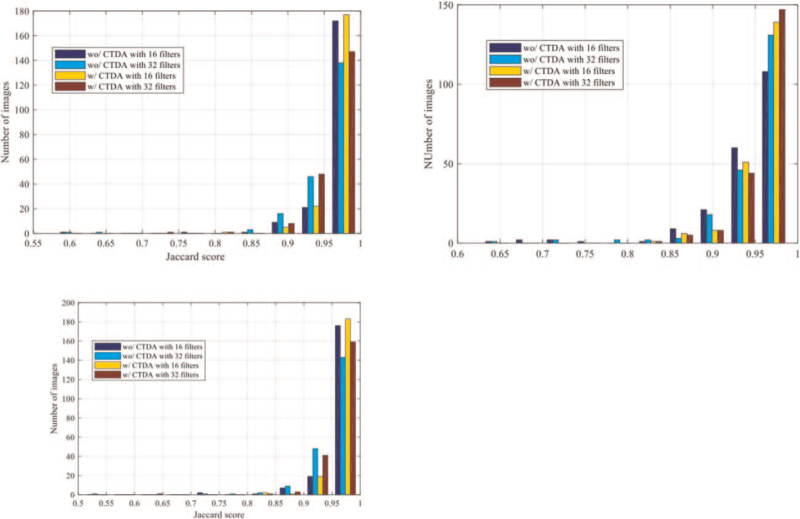
The performance evaluation of the proposed method selection strategy via cross-validation.

Finally, the distributions of the Jaccard score for the test dataset in different rounds of CV are illustrated in Figure 7.

## 4 Discussion

CT is a common examination tool for assessing extent of cancer diseases before starting local or systemic therapy. The information of skeletal muscle obtained concurrently from the CT scan images is of great importance to assist clinicians to draw up the treatment plan. Segmentation and quantification of the ROI of the CT scan images provide the accurate data of the patients’ body composition, not just judged by physicians’ “surgical intuition” or “eyeball test”.^[24]^

Ground truth images are fundamental basis of AI training. Manual depicting of ROI on the CT images for creating ground truth images is a laborious and time-consuming work. Deep convolutional neural networks are heavily reliant on big data to avoid overfitting.^[25]^ Overfitting, occurring when the model performs well on training data but generalizes poorly to unseen data, is one of the primary concerns in machine learning. CV, more training data, feature removal, early stopping, regularization and ensembling are strategies for preventing overfitting. In this study, we employ data augmentation (×5), holdout cross validation (repeated 3 independent rounds) and feature removal (16 features) to prevent the occurrence of overfitting. Overfitting is mild in our study. Data augmentation might be a data-space solution to the problem of limited data commonly encountering in the deep learning of medical images. However, optimal data augmentation should be used prudently. If incremented excessively, overfitting may occur, resulting in a decrease in the training effect. This study focuses on a single original dataset, and expands it into 2 datasets, one with data augmentation operation and the other without. We compare the prediction results to determine whether a larger dataset size can improve the training outcomes. Table 2 summarizes the efficacies of the proposed methodology based on w/ and w/o data augmentation and different feature maps are compared in the experiments. The Jaccard score is approximately 95.0% based on the proposed data augmentation method with only 16 feature maps used in U-Net. The stability and efficiency of the proposed U-Net are verified in the experiments in a CV manner.

**Table 2.** 

Table 2.1 Training set: Performance comparison of the proposed U-Net between different data augmentation strategies terms of Jaccard scores.					
#Features	Policy	CV-1	CV-2	CV-3	Averaged
16	w/o DA	98.8% ± 0.03	98.9% ± 0.03	98.8% ± 0.03	98.8%
16	DA	98.3% ± 0.02	97.5% ± 0.02	98.5% ± 0.02	98.1%
32	w/o DA	98.9% ± 0.03	99.0% ± 0.02	99.1% ± 0.02	99.0%
32	DA	98.4% ± 0.02	98.7% ± 0.02	98.5% ± 0.02	98.5%

Table 2.2 Test set: Performance comparison of the proposed U-Net between different data augmentation strategies terms of Jaccard scores.					
#Features	Policy	CV-1	CV-2	CV-3	Averaged
16	DA	95.0% ± 0.05	95.0% ± 0.04	95.1% ± 0.04	95.0%
32	w/o DA	94.5% ± 0.05	94.6% ± 0.04	94.6% ± 0.05	94.6%
32	DA	95.5% ± 0.03	95.4% ± 0.04	95.2% ± 0.04	95.4%

CV = cross-validation, DA = data augmentation, w/o = without.

The errors in prediction might be attributed to the fuzzy boundary of muscles, which might pose as a problem both for accurate manual labeling in producing the ground truth and AI automatic segmentation. With a high degree of accuracy, the proposed U-Net achieves the state-of-the-art performance and might be potentially applied in clinical practice.

## 5 Conclusions

In the present study, we have developed an improved and simplified U-Net model for CT image segmentation. A data augmentation for paravertebral skeletal muscle CT images has been proposed to improve the data diversity further, leading to auspicious results in terms of the Jaccard score. The efficacies of applying 2 different training sets, one with the original CT scans (616 images) and the other with data augmentation (616 × 5 images) and filter sizes (16 vs 32), are compared. The highest Jaccard score obtained, 95.0%, corresponds to U-Net of a filter size 16 trained with the augmented dataset. The model efficiency, effectiveness, and stability have also been verified in the experiments by CV. As a result, the number of the feature maps of the proposed U-Net can be reduced to 16, as well as 3 CVs still show the superior performance on the separate training sets. In summary, our model shows the high accuracy with a low-complexity requirement, enabling the area and density of the region of the muscle can be easily segmented and derived for semi-automatic diagnosis purposes. Even though several studies about development of delineation methods of the muscle area using AI method including data augmentation and validation are already published,^[11,14]^ further incorporation of information pertinent to patients’ quality of the muscle by HU value changes or consideration of body frame, body mass index, etc shall facilitate the diagnosis of sarcopenia. The strength and originality of our study hinge on the high accuracy (Jaccard score 95.0%) with a low-complexity requirement (16 U-Net feature maps) highly suitable for the clinical application of segmenting the area and density of the muscle region.

This study used the intravenous contrast medium enhanced CT scan. Intravenous contrast medium does have effect on HU of skeletal muscle. However, applying contrast could make the demarcation between muscle and surrounding tissue clear and could be beneficial for initial AI training. Once the AI model, as reported in this paper, is established, AI training of non-contrast medium CT scan images and incorporation of pertinent information (patients’ quality of the muscle by HU value changes, body frame, body mass index, etc) would be the focus of our next phase research.

## Author contributions

**Conceptualization:** Kuen-Jang Tsai, John Y. Chiang.

**Data curation:** Chih-Chun Chang, Lun-Chien Lo, Yu-Jung Huang.

**Formal analysis:** Chih-Chun Chang, John Y. Chiang.

**Investigation:** Kuen-Jang Tsai, Chao-Sung Chang.

**Methodology:** Lun-Chien Lo, Chao-Sung Chang.

**Project administration:** Lun-Chien Lo, John Y. Chiang, Yu-Jung Huang.

**Resources:** Kuen-Jang Tsai, Chao-Sung Chang.

**Software:** Chih-Chun Chang, John Y. Chiang, Yu-Jung Huang.

**Supervision:** Kuen-Jang Tsai.

**Validation:** Chao-Sung Chang.

**Visualization:** Yu-Jung Huang.

**Writing – original draft:** Chih-Chun Chang.

**Writing – review & editing:** Lun-Chien Lo.
